# Worsening of Controlled Attenuation Parameter and Metabolic Profile After HCV Cure in People with HIV as a Sign of Steatosis

**DOI:** 10.3390/v17070906

**Published:** 2025-06-26

**Authors:** Alessia Siribelli, Sara Diotallevi, Laura Galli, Camilla Muccini, Giulia Morsica, Riccardo Lolatto, Tommaso Clemente, Emanuela Messina, Costanza Bertoni, Caterina Uberti-Foppa, Antonella Castagna, Hamid Hasson

**Affiliations:** 1 Department of Infectious Diseases, Vita-Salute San Raffaele University, 20132 Milan, Italy; bertoni.costanza@hsr.it (C.B.); uberti.caterina@hsr.it (C.U.-F.); castagna.antonella@hsr.it (A.C.); 2 Unit of Infectious Diseases, San Raffaele Scientific Institute, 20132 Milan, Italygalli.laura@hsr.it (L.G.); muccini.camilla@hsr.it (C.M.); morsica.giulia@hsr.it (G.M.); lolatto.riccardo@hsr.it (R.L.); clemente.tommaso@hsr.it (T.C.); messina.emanuela@hsr.it (E.M.); hasson.hamid@hsr.it (H.H.)

**Keywords:** HCV, HIV, CAP, DAAs, steatosis, SVR

## Abstract

In HCV-coinfected people with HIV (PWH), there are still conflicting data regarding the long-term metabolic impact of HCV eradication. The aim of the study is to investigate long-term changes in controlled attenuation parameter (CAP) and metabolic profile after sustained virological response (SVR) post-direct acting antivirals (DAAs) in PWH. This is a retrospective observational study including individuals with HIV/HCV coinfection, followed as outpatients at San Raffaele Hospital, who achieved SVR post-DAAs. Individuals were assessed for metabolic parameters before and after the start of DAAs. Univariate and multivariate mixed linear models were calculated to estimate crude mean changes in CAP, metabolic parameters, and weight; slopes were reported with the corresponding 95% confidence intervals (95% CI). Overall, during a median follow-up of 4.02 years (interquartile range, IQR 3.04–4.80), the mean percent increase in CAP was 2.86/year (*p* < 0. 0001), and the mean decrease in stiffness was –4.28 (*p* = 0.003). Additionally, total cholesterol (*p* < 0.0001), high-density lipoprotein (HDL) cholesterol (*p* = 0.001), triglycerides (*p* < 0.0001), glucose (*p* < 0.0001), and Body Mass Index (BMI) (*p* < 0.0001) increased over time. A long-term follow-up in PWH with SVR post-DAAs showed an overall significant increase in CAP and worsening of the metabolic profile, suggesting a higher risk of developing liver steatosis and metabolic alterations over time.

## 1. Introduction

Globally, chronic HCV infection (CHC) affects over 58 million people [1] and can be associated with cirrhosis, hepatocellular carcinoma (HCC), and end-stage liver disease [2]. CHC can also lead to several extrahepatic consequences, including dyslipidemic alterations, hepatic steatosis, insulin resistance (IR), metabolic syndrome (MS), and diabetes (DM) [3]. Studies have shown that different lipogenesis pathways are crucial to the HCV life cycle, particularly during viral entry, replication, and particle assembly [4,5]. Additionally, HCV is implicated in the pathogenesis of liver steatosis [6] and may interfere with insulin signaling pathways, possibly leading to IR [7].

Particularly, HCV genotype 3 has been strongly implicated in the direct induction of hepatic steatosis, independent of traditional metabolic risk factors [7]. This “viral steatosis” is believed to result from the virus’s modulation of lipid metabolism, including upregulation of lipogenic transcription factors such as SREBP-1c and suppression of β-oxidation pathways [8]. Furthermore, HCV core proteins have been shown to impair mitochondrial function and interfere with very-low-density lipoprotein (VLDL) secretion, favoring intracellular lipid accumulation [9]. During HCV clearance with direct-acting antivirals (DAAs), most patients experience a significant increase in circulating total cholesterol (TC) and low-density lipoprotein (LDL-C), which can persist after drug-induced sustained viral suppression (SVR) [10].

People with HIV (PWH) and HCV coinfection, with a global prevalence of nearly 2.3 million individuals [11], show higher rates of HCV persistence and higher viral loads, as well as accelerated progression of liver fibrosis and development of end-stage liver disease compared to HCV-monoinfected individuals [12]. While the condition of hyperlipidemia post-SVR has been confirmed by several studies [10,13], data regarding changes in controlled attenuation parameter (CAP) values in PWH with HCV infection are limited and controversial.

Therefore, the aim of our present study is to evaluate long-term changes in CAP, lipid profile, and the development of diabetes after SVR post-DAAs in PWH.

## 2. Materials and Methods

Our retrospective observational study evaluated PWH with HCV coinfection, who were treated with DAAs for 8, 12, or 24 weeks as outpatients at the Unit of Infectious Diseases of San Raffaele Hospital from September 2013 to July 2022. The inclusion criteria were: SVR12 from end of treatment (EOT); at least one baseline (BL; start of DAAs) value for each parameter considered in the analyses; at least three measurements post-DAA treatment; and no previous diagnosis of DM (defined as at least one value of fasting glucose ≥ 126 mg/dL or the use of anti-diabetic drugs) at BL. Follow-up accrued from the date of SVR12 until the date of the last available visit; the freezing date of data extraction was September 2022.

Demographic, clinical, and laboratory data were collected as part of routine clinical care and recorded in the database of the Unit of Infectious Diseases of San Raffaele Hospital (CSLHIV Cohort), which was approved by the Ethics Committee of San Raffaele Hospital (Milan, Italy; date of approval: 4th December 2017, protocol n. 34). Recorded data are anonymized and managed according to the Good Clinical Practice Guidelines published by the World Medical Association Declaration of Helsinki.

CAP and liver stiffness measurement (LSM) were assessed by vibration-controlled transient elastography (VCTE) using the FibroScan-502 (Echosens, Paris, France^®^), equipped with an M-type or XL-type probe; both parameters were obtained simultaneously to quantitatively evaluate hepatic steatosis [14] and liver fibrosis. Results for CAP and LSM were presented in decibels per meter (dB/m) and kilopascals (kPa), respectively, and were determined under fasting conditions at BL and during follow-up. Ten valid measurements were obtained for each individual, and the median value was considered representative and retained for analysis. Body Mass Index (BMI), fasting glucose, TC, HDL-cholesterol, LDL-cholesterol, and triglycerides were collected at BL and at each follow-up visit. Dyslipidemia was defined as the presence of at least one of the following conditions: total cholesterol >200 mg/dL, triglycerides > 200 mg/dL, HDL cholesterol < 40 mg/dL, LDL cholesterol > 160 mg/dL, or use of fenofibrates and/or statins.

Participants’ characteristics were described as median (interquartile range, IQR) for continuous variables or proportions for categorical variables. Continuous variables were compared using the Kruskal–Wallis test or the Wilcoxon rank-sum test, as appropriate. Differences between proportions were tested using the chi-square or Fisher’s exact test. Incidence rates (IR) were estimated per 100 person-years of follow-up (100-PYFU), using a Poisson regression model.

Mean annual percent changes (slope, β-coefficient) were estimated by univariate linear mixed models with random intercept and slope for all parameters considered in the overall population and according to HCV genotype.

A multivariable linear mixed model on CAP change, with random intercept and slope, was calculated. Covariates potentially influencing CAP change were included in the model; HCV genotype was included as a fixed covariate, while the following characteristics were entered as time-dependent variables: age, BMI, type of Antiretroviral Therapy (ART) regimen [Non-Nucleoside Reverse Transcriptase Inhibitor (NNRTI)-based, Protease Inhibitor (PI)-based, Integrase Strand Transfer Inhibitor (INSTI)-based, other], years on ART regimen, HIV-RNA (<50 vs. ≥50 copies/mL), CD4, diabetes, dyslipidemia, and use of Tenofovir Alafenamide (TAF). Slopes were reported with the corresponding 95% confidence interval (95% CI).

The statistical significance level was set at 5%. All analyses were conducted using SAS statistical software version 9.4 (Statistical Analysis System Inc., Cary, NC, USA).

## 3. Results

### 3.1. Individual Characteristics

Overall, 598 individuals with HIV and HCV were included: at BL, the median age was 53 (49–56), 441 (74%) were male, 150 (25.1%) were MSM, and 302 (50.5%) had a history of intravenous drug use. The median ART duration at BL was 18.3 (11–22) years. At BL, 290 (48.5%) were on an INSTI-based regimen, 141 (23.6%) on a PI-based regimen, 78 (13.2%) on an NNRTI-based regimen, 36 (6.1%) on an INSTI plus NNRTI-based regimen, 39 (6.6%) on an INSTI plus PI-based regimen, and 147 (24.8%) were receiving TAF. At BL, 12 (2%) were on fibrates, and 42 (7%) used statins. HCV genotypes (GT) were distributed as follows: GT1a, 279 (47%); GT1b, 45 (7%); GT2, 23 (4%); GT3, 131 (22%); and GT4, 120 (20%). The most common DAA regimens were sofosbuvir/velpatasvir (48%) and glecaprevir/pibrentasvir (20%), followed by ledipasvir/sofosbuvir (8%), with ribavirin used in 175 cases (29.3%). All individuals achieved SVR12 after treatment with DAAs, and the median follow-up was 4.02 (3.04–4.80) years. BL and follow-up (FU) CAP values were available in 225 individuals. Demographics, clinical features, and ART regimens at BL according to HCV genotype are displayed in Table 1.

### 3.2. Changes in CAP and Other Parameters

**Figure 1:**
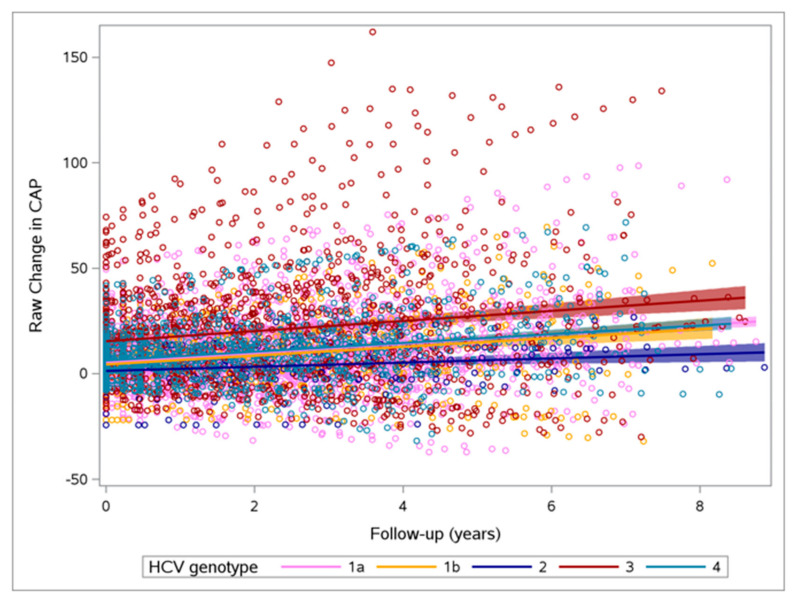
Variation in CAP during follow-up according to HCV genotype.

Overall, a significant increase in CAP was observed: the median CAP at BL was 221 dB/m^2^ (196–254), while the last median value was 242 dB/m^2^ (208–279). The estimated CAP slopes indicated a significant increase in the overall population (β = +2.86, 95% CI: 1.76–3.96, *p* < 0.0001), as well as in the GT1a (β = +2.15, 95% CI: 0.52–3.77, *p* = 0.01), GT1b (β = +5.27, 95% CI: 0.63–9.92, *p* = 0.0265), and GT4 (β = +5.22, 95% CI: 2.85–7.59, *p* < 0.0001) groups (Figure 1). On the other hand, a significant decrease in LSM was observed in the overall group (β = –4.28, 95% CI: –7.16 to –1.40, *p* = 0.003) and in the GT1a group (β = –6.91, 95% CI: –11.19 to –2.62, *p* = 0.0017). No significant differences among subgroups were observed in terms of CAP and LSM changes (Table 2).

In multivariable analysis, the impact of independent factors on CAP changes was explored. After adjustment for years on ART, HCV genotype, current ART type, TAF use, current HIV-RNA, CD4 count, diabetes, BMI, and age, a significant reduction in CAP changes over time (β = −2.85, 95% CI: −5.07 to −0.64, *p* = 0.01) was associated with the presence of dyslipidemia. Moreover, when comparing CAP variation among HCV genotypes, it was found that CAP increased less in individuals with GT3 compared to those with GT4 (β = −4.34, 95% CI: −8.6 to −0.08, *p* = 0.04).

### 3.3. Changes in Metabolic and Anthropometric Parameters

A significant increase was observed after SVR12 in the overall population for TC (β = +1.01, 95% CI: 0.56–1.47, *p* < 0.0001), HDL (β = +1.00, 95% CI: 0.39–1.60, *p* = 0.001), triglycerides (β = +3.05, 95% CI: 1.86–4.23, *p* < 0.0001), fasting glucose (β = +3.40, 95% CI: 3.04–3.76, *p* < 0.0001), and BMI (β = +0.82, 95% CI: 0.61–1.03, *p* < 0.0001). No significant differences were found among genotypes, except for LDL cholesterol (*p* = 0.04), which significantly increased in individuals with GT3 (β = +2.84, 95% CI: 0.59–5.1, *p* = 0.0136) and decreased in those with GT1a (β = –1.5, 95% CI: –3.04 to 0.03, *p* = 0.054). Mean annual percent changes in metabolic and anthropometric parameters overall and according to HCV genotypes are displayed in Table 2.

During follow-up, 20 (3.3%) and 7 (3.1%) new diagnoses of diabetes were recorded in the overall cohort (IR = 0.83/100-PYFU, 95% CI: 0.50–1.28) and among the 225 individuals with CAP data (IR = 0.99/100-PYFU, 95% CI: 0.40–2.04), respectively.

## 4. Discussion

In PWH treated with DAAs for HCV infection, CAP values increased during follow-up after SVR. These findings were consistent with data observed by Chromy et al. [15], who retrospectively analyzed changes in CAP values at 12 weeks of SVR in 138 HIV/HCV coinfected individuals. Moreover, variations in CAP trajectories according to HCV genotype showed a significant increase in CAP in GT4, GT1a, and GT1b, while GT2 and GT3 exhibited a trend toward increased CAP, which was not statistically significant. The modest increase in CAP post-SVR observed in the GT3 group, despite a significant rise in cholesterol and lipid parameters, is consistent with existing knowledge about the unique pathophysiology of GT3 infection. In fact, GT3 is well-established as the HCV genotype most strongly associated with hepatic steatosis [16], often referred to as “viral steatosis,” which is believed to be directly induced by viral mechanisms such as modulation of lipogenesis and lipid oxidation pathways [17]. Moreover, chronic GT3 infection typically results in marked suppression of serum cholesterol and other lipid fractions due to viral interference with hepatic lipid metabolism [18]. Consequently, the observed greater rise in total and LDL cholesterol after SVR in the GT3 group likely support data previously described in literature in both HCV mono-infected people [19] and PWH with HCV coinfection [20], which show a rebound effect following viral clearance, where lipid levels return from a suppressed state toward or above normal values. This may also explain the comparatively smaller increase—or even potential short-term decrease—in CAP in this group, as hepatic steatosis driven by active viral infection may partially resolve after eradication, at least initially. For other genotypes such as GT1a, GT1b, and GT4, our findings showed a significant increase in CAP post-SVR, suggesting ongoing or progressive steatosis development despite viral clearance. These genotypes are generally less directly associated with viral steatosis but may contribute to metabolic alterations through other mechanisms, potentially including host factors [16] or residual liver injury [21]. GT4, in particular, showed one of the highest CAP increases in our study, underscoring the need for further research into genotype-specific liver disease progression. Given the pan-genotypic efficacy of current DAAs, the emphasis in clinical practice has shifted from genotype-directed therapy to broader considerations of long-term liver health and metabolic risk post-SVR. Our study supports this approach by indicating that, despite viral cure, genotype-specific metabolic monitoring remains relevant.

In the multivariable analysis, CAP trajectories were significantly influenced by dyslipidemia. Although both dyslipidemic and non-dyslipidemic individuals showed an increase in CAP trajectories after SVR, this increase was more evident in the non-dyslipidemic subgroup. Individuals with high viral load and persistent HCV replication are at greater risk of developing steatosis due to the activation of inflammatory pathways and the alterations in lipid metabolism [22]. The results of this analysis show a higher increase in CAP trajectories in the non-dyslipidemic subgroup, which may be explained by the combined activity of lipid-lowering drugs (LLDs) and DAAs’ impact on HCV clearance in mitigating lipid alterations and, consequently, reducing the accumulation of fatty acids inside the hepatocytes [23].

The overall sample showed a significant increase in lipid parameters after SVR, particularly TC, HDL, and triglycerides, which is in line with similar data on HCV mono-infected individuals [24]. Within the context of a cohort of PWH coinfected with HCV, the complex interactions between HIV infection, ART, and lipid metabolism require further consideration. Several ART regimens, particularly PIs and some NRTIs, have well-documented effects on lipid profiles, often leading to dyslipidemia [25,26]. These HIV- and ART-related metabolic effects may interact with the changes induced by HCV clearance, contributing to the heterogeneous trajectories observed in hepatic steatosis and lipid parameters among our cohort.

Moreover, a significant and gradual increase in fasting glucose trajectories was observed, which appears in contrast with previous data. Particularly, Chaudhury et al. [27] prospectively evaluated 251 individuals with HCV infection, 31% of whom were PWH. After SVR, with a median follow-up of 28 months, both mono-infected and coinfected individuals did not show significant changes in fasting glucose and HbA1c values. There may be multiple explanations: firstly, the larger cohort and the longer observation period may have given a wider perspective on changes in glucose homeostasis; secondly, PWH are at higher risk of glucose decompensation compared to the general population due to viral and drug-related factors [28,29].

The primary limitation of our study lies in its retrospective design. Secondly, the absence of a control group of HCV mono-infected or HIV mono-infected individuals in our study could be considered an important constraint. Therefore, future prospective studies including appropriate comparison groups will be essential to further clarify the contributions of HIV and HCV infections to long-term metabolic outcomes. In addition, although the analysis was adjusted for the main confounders, we cannot exclude that some unknown or unmeasured confounding factors still remained.

Nonetheless, the long-term follow-up and the larger sample size of this study strengthen the significance of these results and may give a wider perspective on changes in metabolic profile over time, in addition to previous studies. Furthermore, the genotype-specific viral effects on CAP variation and lipid metabolism, alongside the modulatory role of HIV and ART on metabolic parameters, emphasize the complexity of metabolic outcomes following HCV cure in the coinfected population.

## 5. Conclusions

In PWH after HCV eradication, a significant increase in CAP was observed. A worsening metabolic profile in terms of lipid and glucose parameters was also described. These observations may suggest the need for closer monitoring for metabolic syndrome and liver steatosis development or progression in this specific population.

## Figures and Tables

**Table 1:** Summary descriptive table of characteristics at baseline according to HCV genotype.

	1a (N = 279)	1b (N = 45)	2 (N = 23)	3 (N = 131)	4 (N = 120)	*p* Value
Male sex	229 (82.1%)	29 (64.4%)	20 (87.0%)	84 (64.1%)	79 (65.8%)	<0.001
Age	52.0 [46.2; 55.6]	52.2 [50.1; 55.0]	50.8 [44.7; 54.1]	53.7 [51.1; 56.6]	54.2 [51.2; 56.3]	<0.001
Risk Factors:						<0.001
*Hetero*	18 (6.45%)	5 (11.1%)	1 (4.35%)	14 (10.7%)	15 (12.5%)	
*MSM*	104 (37.3%)	7 (15.6%)	15 (65.2%)	10 (7.63%)	14 (11.7%)	
*Other*	40 (14.3%)	8 (17.8%)	3 (13.0%)	24 (18.3%)	18 (15.0%)	
*Ex-drug Users*	117 (41.9%)	25 (55.6%)	4 (17.4%)	83 (63.4%)	73 (60.8%)	
Years (Yrs) of ART	17.3 [7.63; 22.6]	18.4 [14.4; 22.8]	10.5 [6.31; 21.0]	19.1 [14.5; 22.3]	19.4 [13.5; 22.4]	0.047
Yrs with HIV diagnosis	24.3 [11.9; 30.8]	28.5 [19.5; 30.8]	17.6 [10.7; 25.7]	28.1 [21.0; 31.3]	28.0 [20.7; 31.4]	<0.001
Height	175 [169; 179]	170 [164; 176]	174 [169; 180]	170 [166; 175]	172 [167; 178]	0.001
Weight	69.0 [61.0; 77.0]	70.0 [60.0; 76.0]	75.0 [66.5; 80.5]	68.0 [58.0; 79.0]	69.5 [58.0; 77.0]	0.379
Glucose	85.0 [79.0; 93.0]	84.5 [78.2; 95.8]	80.0 [72.0; 87.5]	83.0 [77.0; 93.0]	86.0 [77.0; 94.0]	0.216
Total Cholesterol	168 [140; 190]	167 [149; 194]	191 [152; 210]	157 [119; 186]	165 [145; 186]	0.008
Triglycerides	98.5 [78.8; 133]	110 [90.0; 141]	109 [89.0; 137]	97.0 [77.0; 130]	113 [86.0; 150]	0.093
HDL	47.0 [38.0; 58.0]	44.0 [34.5; 59.8]	49.0 [45.5; 59.5]	46.0 [38.0; 60.0]	47.5 [40.0; 66.0]	0.333
LDL	106 [83.0; 126]	111 [84.5; 130]	137 [94.0; 149]	92.0 [64.0; 122]	97.5 [78.2; 121]	0.013
ALT	62.0 [42.0; 97.0]	64.0 [39.0; 106]	35.0 [29.5; 62.0]	91.0 [49.0; 159]	49.0 [34.0; 74.5]	<0.001
AST	49.0 [35.0; 72.0]	46.0 [35.0; 91.0]	31.0 [25.5; 42.0]	67.0 [45.0; 111]	41.0 [30.0; 65.0]	<0.001
GGT	55.5 [31.0; 108]	67.0 [28.0; 144]	38.0 [23.0; 47.8]	54.0 [32.0; 92.0]	63.0 [34.0; 127]	0.017
PLT	202 [158; 250]	203 [140; 236]	234 [170; 281]	174 [136; 221]	188 [155; 220]	0.001
Albumin	41.9 [39.8; 44.1]	41.3 [38.2; 43.6]	44.0 [42.1; 45.1]	41.8 [39.6; 43.6]	42.8 [40.8; 45.4]	0.049
HIV RNA ≥ 50 cps/mL	24 (8.70%)	6 (13.6%)	0 (0.00%)	6 (4.69%)	10 (8.40%)	0.197
CD4T	643 [470; 839]	624 [365; 878]	679 [542; 836]	670 [468; 868]	712 [518; 878]	0.696
CD8T	789 [580; 1109]	832 [577; 1045]	843 [636; 1096]	800 [573; 1121]	835 [574; 1108]	0.986
FIB_4	1.54 [1.06; 2.37]	1.64 [1.24; 3.19]	1.23 [0.88; 1.69]	2.24 [1.42; 4.16]	1.71 [1.25; 2.35]	<0.001
LSM	7.00 [5.00; 11.0]	8.00 [5.00; 14.0]	6.00 [4.00; 7.00]	8.00 [6.00; 12.2]	7.00 [5.00; 11.2]	0.017
CAP	212 [192; 240]	226 [202; 250]	211 [188; 245]	231 [203; 275]	218 [196; 261]	0.009
Use of fibrates	4 (1.43%)	2 (4.44%)	2 (8.70%)	1 (0.76%)	3 (2.50%)	0.072
Use of statins	19 (6.81%)	4 (8.89%)	2 (8.70%)	3 (2.29%)	14 (11.7%)	0.036
ART regimens:						0.006
INSTI	153 (55.6%)	22 (50.0%)	8 (34.8%)	55 (42.0%)	52 (43.7%)	
INSTI and NNRTI	15 (5.45%)	5 (11.4%)	1 (4.35%)	5 (3.82%)	10 (8.40%)	
INSTI and PI	14 (5.09%)	4 (9.09%)	1 (4.35%)	10 (7.63%)	10 (8.40%)	
NNRTI	41 (14.9%)	2 (4.55%)	7 (30.4%)	12 (9.16%)	16 (13.4%)	
Other	3 (1.09%)	0 (0.00%)	1 (4.35%)	1 (0.76%)	3 (2.52%)	
PI	49 (17.8%)	11 (25.0%)	5 (21.7%)	48 (36.6%)	28 (23.5%)	
TAF-based regimen	76 (27.6%)	10 (22.7%)	6 (26.1%)	25 (19.1%)	30 (25.2%)	0.461

MSM: Men who have sex with Men; AST: aspartate aminotransferase; ALT: alanine transaminase; GGT: gamma-glutamyl transferase; PLT: platelets; CD4T: absolute number of CD4 lymphocytes count; CD8T: absolute number of CD8 lymphocytes count; CAP: controlled attenuation parameter; LSM: liver stiffness measurement; INSTI: integrase strand transfer inhibitors; NNRTI: non-nucleoside reverse transcriptase inhibitors; PI: protease inhibitors; TAF: tenofovir alafenamide.

**Table 2:** Mean annual percent changes (95% Confidence Interval) of CAP and metabolic parameters.

Parameter	Overall	GT1a	GT1b	GT2	GT	GT4	*p* Value Difference Between Groups
Total Cholesterol	1.09 (0.56; 1.47) *p* value = <0.0001 N = 540	0.79 (0.12; 1.46) *p* value = 0.0203 N = 253	1.46 (−0.13; 3.05) *p* value = 0.0728 N = 40	0.59 (−1.67; 2.86) *p* value = 0.6085 N = 21	1.9 (0.96; 2.84) *p* value = <0.0001 N = 120	0.75 (−0.3; 1.81) *p* value = 0.1606 N = 106	0.3533
LDL	−0.26 (−1.34; 0.82) *p* value = 0.6360 N = 376	−1.5 (−3.04; 0.03) *p* value = 0.0547 N = 181	0.36 (−3.35; 4.06) *p* value = 0.8503 N = 25	−0.55 (−5.6; 4.51) *p* value = 0.8322 N = 15	2.84 (0.59; 5.1) *p* value = 0.0136 N = 86	0.47 (−2.16; 3.1) *p* value = 0.7252 N = 69	0.0408
HDL	1.00 (0.39; 1.61) *p* value = 0.0013 N = 397	0.51 (−0.38; 1.39) *p* value = 0.2603 N = 189	2.02 (0.03; 4) *p* value = 0.0467 N = 28	1.75 (−1.22; 4.73) *p* value = 0.2482 N = 15	1.9 (0.64; 3.16) *p* value = 0.0031 N = 90	0.53 (−0.89; 1.95) *p* value = 0.4650 N = 75	0.3076
Triglycerides	3.05 (1.87; 4.24) *p* value = <0.0001 N = 537	2.79 (1.03; 4.54) *p* value = 0.0019 N = 250	4.87 (0.91; 8.84) *p* value = 0.0160 N = 40	2.07 (−3.59; 7.73) *p* value = 0.4731 N = 21	1.88 (−0.51; 4.28) *p* value = 0.1238 N = 120	4.41 (1.63; 7.19) *p* value = 0.0019 N = 106	0.5852
Weight	0.79 (0.59; 0.99)*p* value = <0.0001 N = 545	1.01 (0.72; 1.29) *p* value = <0.0001 N = 255	0.73 (−0.01; 1.47) *p* value = 0.0529 N = 39	0.09 (−0.83; 1.02) *p* value = 0.8414 N = 22	0.43 (0.02; 0.84) *p* value = 0.0406 N = 118	0.88 (0.42; 1.33) *p* value = 0.0002 N = 111	0.1137
Stiffness	−4.28 (−7.16; −1.41) *p* value = 0.0036 N = 287	−6.91 (−11.19; −2.62) *p* value = 0.0017 N = 126	−2.01 (−13.22; 9.19) *p* value = 0.7236 N = 19	−2.25 (−15.49; 11) *p* value = 0.7384 N = 10	0.67 (−5.09; 6.42) *p* value = 0.8197 N = 70	−5.5 (−11.95; 0.95) *p* value = 0.0940 N = 62	0.3203
BMI	0.82 (0.61; 1.03) *p* value = <0.0001 N = 541	1.02 (0.71; 1.32) *p* value = <0.0001 N = 252	0.72 (−0.07; 1.5) *p* value = 0.0739 N = 39	0.09 (−0.89; 1.08) *p* value = 0.8518 N = 22	0.49 (0.05; 0.93) *p* value = 0.0280 N = 117	0.97 (0.48; 1.45) *p* value = <0.0001 N = 111	0.1816
Glucose	3.40 (3.04; 3.76) *p* value = <0.0001 N = 574	3.36 (2.83; 3.88) *p* value = <0.0001 N = 268	4.14 (2.92; 5.36) *p* value = <0.0001 N = 42	1.38 (0.35; 3.10) *p* value = 0.1181 N = 23	3.43 (2.69; 4.18) *p* value = <0.0001 N = 126	3.62 (2.77; 4.48) *p* value = <0.0001 N = 115	0.1381
CAP	2.86 (1.76; 3.96) *p* value = <0.0001 N = 225	2.15 (0.52; 3.77) *p* value = 0.0101 N = 100	5.27 (0.63; 9.92) *p* value = 0.0265 N = 12	1.49 (−3.54; 6.52) *p* value = 0.5576 N = 7	1.84 (−0.33; 4) *p* value = 0.0956 N = 53	5.22 (2.85; 7.59) *p* value = <0.0001 N = 53	0.1509

LDL: low-density lipoprotein; HDL: high-density lipoprotein; BMI: Body Mass Index; CAP: Controlled Attenuation Parameter.

## Data Availability

The data presented in this study are available at the request of the corresponding author due to privacy and ethical restrictions.

## Abbreviations

The following abbreviations are used in this manuscript:

CHC = Chronic hepatitis C; PWH = People with HIV; CAP = Controlled attenuation parameter; SVR = Sustained virological response; DAAs = Direct acting antivirals; GT = Genotype; HCC = Hepatocarcinoma; IR = Insulin resistance; MS = Metabolic syndrome; DM = Diabetes mellitus; VLDL = Very low density protein; SREBP = Sterol-regulatory-element-binding-protein; EOT = End of therapy; TC = Total cholesterol; ART = Antiretroviral therapy; BL = Baseline; LSM = Liver stiffness measurement; VCTE = Vibration-controlled transient elastography; LLD = Lipid-lowering drugs
